# Deficiency in the Screening Process of Fabry Disease: Analysis of Chronic Kidney Patients Not on Dialysis

**DOI:** 10.3389/fmed.2021.640876

**Published:** 2021-02-09

**Authors:** Yuri Battaglia, Fulvio Fiorini, Cristiano Azzini, Pasquale Esposito, Alessandro De vito, Antonio Granata, Alda Storari, Renzo Mignani

**Affiliations:** ^1^Division of Nephrology and Dialysis, St. Anna University Hospital, Ferrara, Italy; ^2^Division of Nephrology and Dialysis, “Santa Maria della Misericordia” Hospital, Rovigo, Italy; ^3^Department of Neuroscience and Rehabilitation, Ferrara University Hospital, Ferrara, Italy; ^4^Division of Nephrology, Dialysis and Transplantation, Istituto di Ricovero e Cura a Carattere Scientifico (IRCCS) Ospedale Policlinico San Martino, Genoa, Italy; ^5^Department of Internal Medicine, University of Genoa, Genoa, Italy; ^6^Division of Nephrology and Dialysis, “Cannizzaro” Hospital, Catania, Italy; ^7^Division of Nephrology and Dialysis Department, Infermi Hospital, Rimini, Italy

**Keywords:** Fabry disease, chronic kidney disease, screening, prevention, lysosomal disorders

## Abstract

Fabry Disease (FD), a rare and progressive, X-linked lysosomal storage disorder, is caused by mutations in the α-galactosidase A (GLA) gene which leads to enzymatic deficiency of GLA. Misdiagnosed and undiagnosed FD cases are common for the variable FD phenotype, ranging from asymptomatic and/or impairment of single organs, which is typically seen in females and in patients with late-onset mutation, to multiple organ disease, which is frequently found in males with classic GLA mutation. Consequently, for an early diagnosis and an efficient treatment of FD, three different strategies of screening, new-born screening, high-risk screening and familiar screening, have been conducted. However, most of FD screening in the CKD population has been carried out in hemodialysis patients and kidney transplant recipients, for whom the renal damage is already irreversible, so the effectiveness of enzymatic replacement therapy is limited and delayed therapeutic intervention results in worse long-term outcomes. This review investigates the actual strategies of screening initiatives for the identification of FD, examining in detail those performed in CKD patients not on dialysis.

## Introduction

Screening is considered one of the main processes to detect healthy patients affected by latent or early symptomatic stages of the disease (1). It allows an early diagnosis and an effective treatment of the disease, reducing the cost of medical care and improving the quality of life.

According to the classic Wilson and Jungner criteria^1^, this procedure takes into account whether the disease is adequately understood and is characterized by specific findings, including a delay between the detection of risk factors and the onset of the disease.

Due to the fact that chronic kidney disease (CKD) occurs asymptomatically and is clinically silent in early stages, large-scale national screening programs (2) have been only conducted in some countries, showing more than 10% presence of markers for renal damage (3).

However, a global awareness campaign, promoted by international and national societies of nephrology to reduce the impact of CKD, confirmed that risk factors, such as high blood pressure, were found in roughly 10% of both adult and adolescent populations (4–6).

Although the etiology of CKD is primarily due to diabetes and hypertension (7–9), a considerable proportion of unknown CKD cases are caused by undetected genetic diseases, including Fabry Disease (FD).

This review analyzes the recent developments on different approaches of screening FD programs with a specific focus on those which were performed on not dialysis-dependent chronic kidney disease (NDD-CKD) patients.

### Pathogenesis and Clinical Manifestation of Fabry Disease

Over the last 20 years, an increasing amount of the Nephrology community's attention has been captured by FD, a hereditary, X-linked, lysosomal storage disease (10). It is caused by deficiency of α-galactosidase A (GLA) and the gene encoding GLA is located on long arm of X-chromosome (Xq21-22) (11). The enzyme deficiency leads to the accumulation of glycosphingolipids, mainly globotriaosylceramide (Gb3) and globotriaosylsphingosine (lyso-GB3), in lysosomes of skin, eye, kidney, heart, brain, and the peripheral nervous system (12–14). FD is a progressive disease that worsens with the age. Clinical manifestations ensue during childhood or early adolescence with angiokeratomas, lenticular and corneal opacity, microalbuminuria or proteinuria, gastrointestinal pain, recurrent fever, alterations of thermoregulation (i.e. hypohidrosis or anhidrosis) and in the peripheral nervous system (15).

In adulthood, patients show manifestations of FD in the cardiac, cerebrovascular, and renal systems (16–18). In particular, renal impairment is one of most pervasive signs of FD (19). Firstly, a glomerular hyperfiltration, likely due to the activation of the sympathetic nervous system, increased oxidative stress and inflammatory cytokines, associated with a mesangial cell proliferation occurs (20). Later, early morphologic findings of impaired renal function, such as the development of a focal and segmental glomerulosclerosis and vascular changes, are observed (21). With the progression of the disease, proteinuria and progressive renal failure leading to dialysis (ESRD) appears in ~50% and 20% of male and female FD patients, respectively (22).

This clinical picture, which is considered the classic type of FD, leads to death of male patients during the fourth or fifth decade of life (23). By contrast, female patients commonly show milder involvement due to random inactivation of the X-chromosome, determining the development of organs which are chimeras of normal and affected cells (24).

Besides the classic type, milder and late-onset forms of FD have been described as atypical variants. They are characterized by non-specific symptoms and/or involvement of single organ, also defined cardiac and renal variants (25). Therefore, diagnosis of FD in patients with atypical variants is often difficult and it is generally made in the more advanced stages of disease (26).

However, early diagnosis is compulsory in patients with FD since early therapeutic interventions, such as enzymatic replacement therapy and chaperone therapy, significantly stabilize the disease and prevent the progression of renal, cerebrovascular and cardiovascular complications (27–29).

### Screening in CKD Patients Not on Dialysis

Unfortunately, the true prevalence of FD is not well defined. Initially, underestimated prevalence (1:117,000–1:833,00) of FD was found in general population (30–32), mainly because of the lack of clear genotype-phenotype correlation. Subsequently, large new-born and high-risk screening studies showed higher FD prevalence than previously expected. A recent re-analysis of studies for pathogenic GLA mutation, conducted on high-risk populations, showed that the prevalence of FD in 5,491 (4,054 males and 1,437 female) patients with no definitive cause of left ventricular hypertrophy was 0.94% in males and 0.90% in females and among the 5,978 (3,904 males and 2,074 females) young patients with unexplained stroke, 0.13% were males and 0.14% were females (33) (more details are described in the next paragraphs). Nevertheless, few researchers have addressed the problem of screening in NDD-CKD population to detect patients affected by FD, determining its prevalence.

Although the ERBP guidelines (34), in 2013, recommended screening for FD in NDD-CKD patients, no study had been published to support this ungraded statement at the time. However, a singular study performed by Andrade et al. (35), screened 499 renal male patients (of which 141 not on dialysis, 159 on hemodialysis, 59 on peritoneal dialysis, and 138 with a kidney transplant) by plasma GLA assay in a large Canadian province (population ~4.25 million). No new FD cases were found despite the fact that multiple racial populations, including 71 white and 26 Asian, were exanimated.

For the first time in 2016, Turkmen et al. (36) showed in their TURKFAB Study the FD prevalence was 0.95% (3/313) in stage 1–5 NDD-CKD patients with unknown etiology of 10 Turk centers. Furthermore, 8 other patients with both FD and CKD were identified from two index patients, for a total of 11 cases of 386 screened people.

To date, only four FD screening studies reported data about CKD patients, the TURKFAB Study (T1) described above (36), one conducted in the Aegean region of Turkey (T2) (37), one in Taiwan (T3) (38) and, recently, one in Canada (C1) (39) (Table 1).

**Table 1:** Characteristics of Fabry disease screening studies in chronic kidney disease patients not on dialysis.

**Study**	**Turkmen et al.** **(T1)**	**Yeniçerioglu et al.** **(T2)**	**Lin et al.** **(T3)**	**Auray-Blais et al.** **(C1)**
Overall screened	313	1453	1012	397
Males screened	167	797	1012	279
Females screened	146	656	0	118
Screening test	Enzymatic assay	Enzymatic assay	Enzymatic assay	Gb3 and Lsyso-Gb3 urinary assay
Confirmation test	DNA test	DNA test	DNA test	Enzymatic assay or/and DNA test
FD cases, *n*	11	3	6	0
FD prevalence, %	0.95	0.2	0.59	0
Mutation classic (*n*)	p.N34H (8) p.F229V (2) p.358delE (1)		p.T410A (1) p.G138E (1)	
Mutation atypical (*n*)		A143T variant (1) D313Y variant (2)	c.639 + 919G > A (3) p.P210S (1)	
GFR, ml/min	87 (23–126)^*^	33.9 ± 3.9^**^	50.5 ± 28.0^**^	
Proteinuria	0.26 (0.07–5.6)^*^g/g^***^	50 ± 6.2^**^ mg/24 h	2.0 ± 2.4^**^ g/24 h	
FD cases with cardiovascular complications, *n*	2	2	4	
FD cases with cerebrovascular complications, *n*	0	0	2	
FD cases with other signs or symptoms, *n*	9	2	2	

* Median (IQR);

** Mean ± SD;

*** *Spot urine total protein/creatinine ratio; FD: Fabry Disease*.

In a total of 3,175 (313 in T1; 1,453 in T2; 1,012 in T3; 397 in C1) follow-ups of stable CKD (3–5 stages in T2 and C1; 1–5 stages in T1 and T3) patients in 23 centers (10 in T1; 7 in T2; 2 in T3; 4 in C1), the prevalence ranged between 0% and 0.95%, of which 0.4–1.8% and 0.0–1.0% in 2,255 males and 920 females, respectively.

Surprisingly, a slightly lower average age of 16 male cases (47 years) compared with 4 female cases (49 years) was identified. This finding is in contradiction with the suggestion of performing FD screening in men under 50 years and in all ages for women, according to ERBP working groups (34). Furthermore, stratifying the cases by type of GLA mutation, 9 males with classic mutations were younger than 7 males with atypical mutations (35 and 62 years, respectively). This result seems to indicate that FD screening should be conducted in men of all ages to identify not only the classical GLA mutations, but also the late-onset GLA mutations.

All CKD patients were screened by analyzing GLA activity in dried venous blood spots (DBS) and, when enzyme activity was lower than 1.2 micromole/L/h, the GLA gene was sequenced, except for 397 patients of C1 study in which an innovative method, the dosage of Gb3 and lyso-GB3 in dried urine spots, was used. Although the enzymatic activity is normal or slightly lower in roughly 30% of FD females, only one study (T3) excluded female to avoid false-negative results. Accordingly, the prevalence of FD could be underestimated in approximately one-third (females: 146 in T1; 656 in T2) of screened CKD patients. Furthermore, in the Canadian study (C1) 43% of suspected FD patients, screened with the urine test, died in the course of study before an enzymatic or genetic test was performed.

Ancillary, Nakagawa et al. (40) screened 2,325 Japanese patients affected by typical cardiac, renal, or neurological FD complications, including 374 (16.1%) subjects with proteinuria of which 129 (34.5%) were female and 245 (65.5%) were male. Of ten patients with GLA mutations (6/10 were pathogenic), two had a nephrotic range proteinuria, namely a 37-year-old female with juvenile minor ischemic stroke and a 56-year-old male with CKD. Although data of renal function among the screened are missing, this study highlights the importance of investigations not only in CKD patients with unknown causes, but also in the presence of proteinuria, an early finding of renal damage. Furthermore, the results of the ongoing study, HISTOFAB^2^ (ClinicalTrials.gov identifier: NCT03869554), might improve the understanding of the FD screening benefits also in CKD patients with undetermined etiology after a renal biopsy.

**Figure 1:**
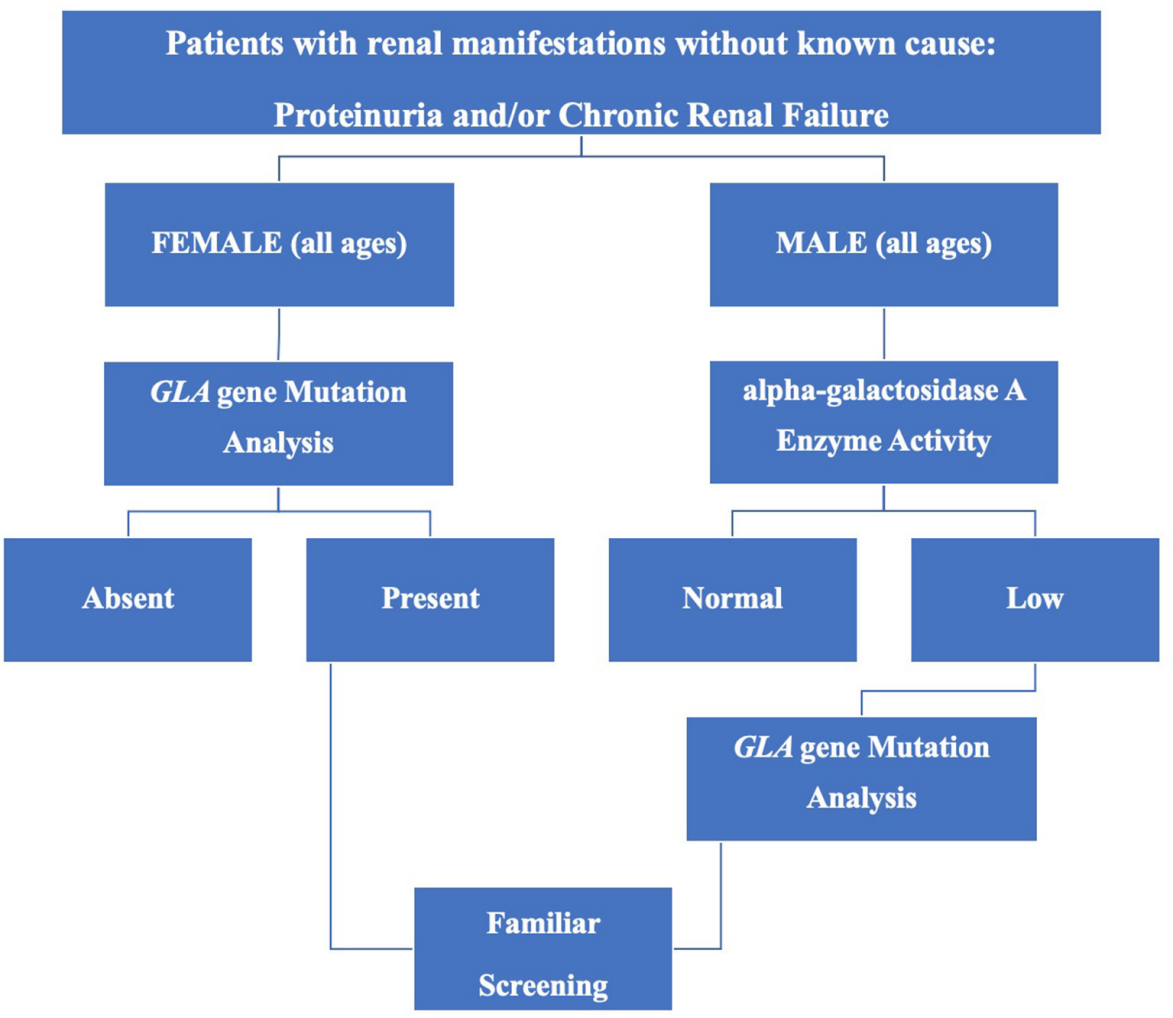
Screening for Fabry disease in chronic kidney disease patients not on dialysis.

Finally, in order to help identify suspected FD patients, efficient and convenient screening protocols have been proposed (34, 41) and on the basis of available data, a comprehensive flow-chart is shown in Figure 1.

### High-Risk Screening in Dialysis Patients and Kidney Transplant Recipients

In CKD population, most screening studies have been carried out in patients receiving hemodialysis treatment or after renal transplantation (42). Doheny et al. (33) showed an FD prevalence of 0.21% in 23,954 male dialysis patients (of these 66% had the classic phenotype and 34% had late-onset phenotype) and 0.15% in 12,866 female dialysis patients (of these 68.4 and 31.6% had classic and late-onset phenotype, respectively) among the 27 reviewed studies. Also, 5 (0.24%) pathogenic mutations among the 2,031 KTR males were reported; and, none among the 1,043 KTR females was found. By contrast, other recent studies demonstrated a higher percentage of GLA mutations in females compared to males (43). Notably, of the 265 KTR screened, Veroux et al. (44) identified six patients (one male and five female) with a pathogenic GLA mutation (2.3%) and one female patient with a benign variant of GLA mutation (0.3%). This higher incidence (2.3%) of KTRs with undetected FD could be explained by the presence of many clusters of FD in the population and by the homogeneity of cohort.

Finally, high-risk screening in KTRs and dialysis patients is able to facilitate the detection of unrecognized FD patients even though the CKD and FD stages in these patients are remarkably advanced with a high risk of adverse outcomes and a poor efficacy of delayed treatment.

### Family Screening

In order to increase the diagnostic rate of FD, currently, another different approach, the family screening, is suggested (45). It is crucial for X-linked transmission of FD. Indeed, for an index case with FD, a pedigree on average three generations is usually performed and approximately five family members are identified (46). Secondary analysis of some studies reported that the prevalence of FD among relatives of FD patients was about 2%, with higher percentage in males (47). By contrast, in a recent family screening study (48), of 1,214 relatives of 71 CKD patients with FD, it was found that the majority of the 115 (9.74%) family members with a GLA mutation were women (66.1%) and under 44 of age (74%). This conflicting data is not surprising since most of the screenings were performed with GLA activity dosage and/or with female exclusion. It may be related to lower costs of enzymatic analysis, which is adequate for screening diagnosis in males, compared to genetic sequencing, which is mandatory in females that often have normal GLA activity because of lyonization (22). However, the GLA genotyping should be performed in the patients screened with null or low GLA activity, to distinguish among the classic, the late-onset, and the benign mutations (49).

On the other hand, other studies reported only the number of cases, ranging between 8 and 24 cases (50–53). Recently, in a kidney transplant (KTR) screening study (44), seven FD probands' relatives were evaluated, finding ten females (five sisters, four daughters, and one nephew) and two males (one brother and one son) with pathogenic GLA mutation. 5/12 FD relatives received an early treatment before irreversible organ damage. This data confirms that the family screenings are able to delay the progression of FD and lead to better long-term outcomes.

### New-Born Screening

In Northern Italy, Spada et al. conducted the first screening study to detect FD in the newborns, finding an incidence of GLA deficiency at 1/3,100. Most of identified GLA mutations were likely to result in atypical phenotype (54). Subsequently, several new-born screening studies confirmed higher prevalence of both FD classic males (ranging from 1:22,000 to 1:40,000) and FD atypical phenotype (1:1,000–1:3,000 in males and 1:6,000–1:40,000 in females) compared to other strategy of screening (55, 56).

However, according to the European Renal Best Practice (ERBP) (34) and the KDIGO controversies conference (45), the new-born screening to diagnose FD patients is not justified in general population for various clinical and ethical issues, including the high prevalence of atypical forms observed in the infants, as reported above. Although Hsu et al. highlighted that patients with late-onset phenotype are likely to have a silent progression of irreversible organ damage (57), the optimal time for screening late-onset variants remains debated. The best strategy to avoid irreversible damage in FD adults is an early diagnosis and effective treatment, but only a better characterization of the natural history of atypical FD forms could make the use of newborn screening advantageous.

## Conclusion

Although expert working groups strongly recommend improvement in the screening process, few FD screening studies have been published for NDD-CKD patients and the data of FD prevalence is inconclusive. However, the importance of early detection of both CKD and FD is fundamental to start efficient treatment and to reduce the rate of morbidity and mortality. Clinicians should become conscious of the fact that FD needs to be taken into account in the differential diagnosis of CKD patients with unknown etiology even without other FD manifestations. Further multicenter systematic large-scale screening studies must be encouraged to assess the value of this screening strategy and to determine the true prevalence of FD in NDD-CKD patients.

## Author Contributions

YB: conceptualization. CA and AD: investigation. YB and PE: writing and editing. FF and AG: supervision. AS and RM: validation. All authors have read and agreed to the published version of the manuscript.

## Conflict of Interest

The authors declare that the research was conducted in the absence of any commercial or financial relationships that could be construed as a potential conflict of interest.

## Footnotes

^1^Available online at: https://apps.who.int/iris/bitstream/10665/37650/17/WHO_PHP_34.pdf (accessed December 1, 2020).

^2^Available online at: https://www.clinicaltrials.gov/ct2/show/study/NCT03869554?term=screening&cond=Fabry$+$Disease&draw=2&rank=5 (accessed December 1, 2020).
